# MicroRNAs show diverse and dynamic expression patterns in multiple tissues of *Bombyx mori*

**DOI:** 10.1186/1471-2164-11-85

**Published:** 2010-02-02

**Authors:** Shiping Liu, Song Gao, Danyu Zhang, Jiyun Yin, Zhonghuai Xiang, Qingyou Xia

**Affiliations:** 1 The Key Sericultural Laboratory of Agricultural Ministry, College of Biotechnology, Southwest University, Chongqing 400715, PR China; 2 Institute of Agricultural and Life Sciences, Chongqing University, Chongqing, 400030, PR China

## Abstract

**Background:**

MicroRNAs (miRNAs) repress target genes at the post-transcriptional level, and function in the development and cell-lineage pathways of host species. Tissue-specific expression of miRNAs is highly relevant to their physiological roles in the corresponding tissues. However, to date, few miRNAs have been spatially identified in the silkworm.

**Results:**

We establish for the first time the spatial expression patterns of nearly 100 miRNAs in multiple normal tissues (organs) of *Bombyx mori *females and males using microarray and Northern-blotting analyses. In all, only 10 miRNAs were universally distributed (including bmo-let-7 and bmo-bantam), while the majority were expressed exclusively or preferentially in specific tissue types (e.g., bmo-miR-275 and bmo-miR-1). Additionally, we examined the developmental patterns of miRNA expression during metamorphosis of the body wall, silk glands, midgut and fat body. In total, 63 miRNAs displayed significant alterations in abundance in at least 1 tissue during the developmental transition from larvae to pupae (e.g., bmo-miR-263b and bmo-miR-124). Expression patterns of five miRNAs were significantly increased during metamorphosis in all four tissues (e.g., bmo-miR-275 and bmo-miR-305), and two miRNA pairs, bmo-miR-10b-3p/5p and bmo-miR-281-3p/5p, showed coordinate expression.

**Conclusions:**

In this study, we conducted preliminary spatial measurements of several miRNAs in the silkworm. Periods of rapid morphological change were associated with alterations in miRNA expression patterns in the body wall, silk glands, midgut and fat body during metamorphosis. Accordingly, we propose that corresponding ubiquitous or tissue-specific expression of miRNAs supports their critical roles in tissue specification. These results should facilitate future functional analyses.

## Background

MicroRNAs (miRNAs) are an extensive class of small (~22 nucleotides) regulatory RNAs found in a wide range of eukaryotic organisms and viruses [1,2]. Increasing evidence shows that miRNAs function in several biological processes, including development, cellular differentiation, proliferation, metabolism and apoptosis [1,3-5]. Single miRNAs may regulate hundreds of different target genes at the post-transcriptional level, and extensively control more than 30% of animal genes [3,6]. Plant miRNAs regulate the expression of other genes by cleaving perfect or nearly perfect complementary sites within the transcribed regions [7], whereas animal miRNAs repress the translational expression of genes by partially binding to the complementary target sites at 3'untranslated regions (3'UTRs) [8,9]. The first members of the miRNA family discovered in invertebrates, lin-4 and let-7, are expressed in *Caenorhabditis elegans *at distinct stages of development and regulate the timing of larval transition through cell-fate decisions [8,10]. In insects, bantam miRNA regulates cell proliferation and death by targeting the apoptosis gene *hid *(*wrinkled*) [11]. *Drosophila *miR-14 is implicated in fat metabolism, stress resistance and cell death [12]. Interestingly, most of these well-characterized miRNAs are highly conserved between invertebrates and vertebrates, resulting in maintenance of their regulatory functions across species [13].

The tissue-specific expression patterns of miRNAs observed in many species [14-17] are considered a prerequisite for specifying and maintaining tissue identity [18]. Moreover, tissue- and cell-specific expression patterns are directly associated with the physiological functions of miRNAs. For instance, miR-1 is specifically expressed in cardiac and skeletal muscle precursor cells, and regulates cardiomyocyte proliferation in vertebrates [19]. In *Drosophila*, miRNA-1 is strongly expressed throughout the mesoderm of early embryos and subsequently in somatic, visceral, and pharyngeal muscles and the dorsal vessel, and functions in the post-mitotic growth of larval muscle [20]. miR-122 is an abundant liver-specific miRNA [21], which facilitates replication of the hepatitis C viral RNA in humans [22] and regulates cholesterol and fatty-acid metabolism [23,24]. Establishment of the tissue-specific distribution of miRNAs in these species offers critical insights into the roles of corresponding miRNAs in tissue specification and cell lineage decisions.

*B. mori*, a characteristic representative of Lepidoptera, has long been used as a model for lepidopteran biology [25]. A number of silkworm miRNAs have been experimentally identified and temporally characterized [26-30]. However, only the spatial expression patterns of let-7 have been extensively characterized in the silkworm to date [29]. In this study, we established the distribution of silkworm miRNAs in fifth-instar day 3 larvae and their spatiotemporal expression patterns in the body wall, silk glands, midgut and fat body during metamorphosis.

## Results and Discussion

### Identification of miRNAs in multiple tissues of fifth-instar day 3 larvae

To determine the global spatial expression patterns of miRNAs in silkworm, we designed a DNA oligonucleotide-based microarray examining 92 unique miRNAs with 106 antisense probes [30]. To verify results, all probes for miRNAs and controls were printed in triplicate on two parallel blocks. The microarray raw data and processed files have been deposited in the NCBI Gene Expression Omnibus [31] and are accessible through GEO Series accession number GSE18039 http://www.ncbi.nlm.nih.gov/geo/query/acc.cgi?acc=GSE18039. To make the inter-slide signals comparable, all net signals of fifth-instar day 5 larvae were normalized on the basis of U6 and 5 S rRNA signals. All internal and external controls were deleted after normalization. In all, only 10 miRNAs (No.37-46), including bmo-let-7, bmo-miR-34b and bmo-bantam, were ubiquitously expressed in all tissues examined, whereas individual tissues were enriched for distinct sets of miRNAs (Table 1). For example, strong expression signals of miR-124 (No.1) and miR-263b (No.4) were specifically detected in the head. Similarly, miR-124 is restricted to the central nervous system of the fruit fly [17] and only expressed in the brain of mouse [32]. The *Drosophila *orthologue of bmo-miR-7 has been identified in the nervous system, and may be related to segmentation, sensory organ development, and Notch signal transduction [17]. In the silkworm, miR-7 (No.10) was expressed in the head and gonads in both sexes, as well as in the body wall in males. Three miRNAs, miR-288 (No.14), miR-278 (No.16) and miR-13a (No.17) were solely detected in the head, body wall and malpighian tubules. A previous study shows that miR-274 is expressed predominantly in a single anterior stripe in *Drosophila *blastoderm embryos roughly corresponding to the intercalary segment [17]. In the silkworm, miR-274 (No.15) expression was restricted to the silk glands and malpighian tubules. miR-1 (No.22) was most strongly detected in the head and body wall, followed by the midgut, fat body and the malpighian tubules, but was undetectable in the silk glands, gonads and hemocytes. miR-279 (No.25) and miR-307-5p (No.30) were expressed in the head, body wall, fat body, ovaries, hemocytes and malpighian tubules.

**Table 1:** Spatial distribution of miRNAs in fifth-instar day 3 larvae

No.	miRNA	Probe sequence (5'-3')	HD	BW	ASG	PSG	MG	FB	OV	TE	HC	MT	Nor
1	miR-124	TAAGGCACGCGGTGAATGCCAAG	+	+									+
2	miR-1497	GCCCACCACCTGCTGCATGTA	+										+
3	miR-286	AGCACGAGTGTTCGGTCTAGTCA		+								+	NA
4	miR-263b	CTTGGCACTGGGAGAATTCAC	+										+
5	anti-miR-276-5p	AGCGAGGTATAGAGTTCCTACGT		+								+	NA
6	miR-5	CATATCACAACGATCGTTCCTTT		+								+	NA
7	miR-282	ACAGACAAAGCCTAGTAGAGGCTAGATT		+								+	NA
8	miR-29b	GCACTGAATTCGAATGGTGCTA		+						+		+	+
9	miR-283	CCCAGAATTACCAGCTGATATTTA		+			+					+	+
10	miR-7	ACAACAAAATCACTAGTCTTCCA	+	+					+	+			+
11	miR-9a	TCATACAGCTAGATAACCAAAGA	+	+					+				+
12	miR-228	CCGTGAATTCTTCCAGTGCCATT	+	+	+	+	+			+		+	+
13	miR-92	GGACTCCCTACTAGAGTCAATTT		+					+			+	+
14	miR-288	CATGAAATGAAATCGACATGAAA	+	+								+	+
15	miR-274	ATTACCCGTTAGTGTCGGTCACAAAA			+	+						+	+
16	miR-278	AAACGGACGAAAGTCCCACCGA	+	+								+	NA
17	miR-13a	CCACATCAAAGTGGCTGTGATA	+	+								+	+
18	miR-275	CGCGCGCTACTTCAGGTACCTGA	+	+					+		+	+	+
19	miR-10b-3p	ACCTCTCTAGAACCGAATTTGT	+	+			+					+	+
20	miR-10b-5p	ACAAATTCGGATCTACAGGGT	+	+			+					+	+
21	miR-133	CTACAGCTGGTTGAAGGGGACCAAATG	+	+				+				+	+
22	miR-1	CTCCATACTTCTTTACATTCCA	+	+			+	+				+	+
23	miR-277	TGTCGTACCAGATAGTGCATTTA	+	+	+	+						+	NA
24	miR-iab-4-5p	TCAGGATACATTCAGTATACGT		+			+	+	+	+		+	+
25	miR-279	TCAATGAGTGTAGATCTAGTCA	+	+				+	+	+	+	+	+
26	miR-281-3p	ATAAAGAGAGCAACTCCATGACA	+	+	+	+	+					+	+
27	miR-281-5p	ACTGTCGACGGATAGCTCTCTT	+		+	+	+					+	+
28	miR-252	CCTGCGGCACTAGTACTTAGGAA	+	+				+	+	+		+	+
29	miR-307-3p	TCACAACCTCCTTGAGTGAG											
30	miR-307-5p	ACATCACACCCAGGTTGAGTGAGT	+	+				+	+		+	+	NA
31	miR-12	ACCAGTACCTGATGTAATACTCA	+	+	+	+	+					+	NA
32	miR-184-3p	GCCCTTATCAGTTCTCCGTCCA	+	+			+	+	+	+	+	+	+
33	miR-31a	ACAGCTATGCCGACTTCTTGCCT	+	+	+	+	+	+	+	+		+	+
34	miR-305	CAGAGCACCTGATGAAGTACAAT	+	+	+	+	+		+	+	+	+	+
35	miR-276-3p	AGAGCACGGTATGAAGTTCCTA	+	+	+	+	+	+	+		+	+	+
36	miR-276-5p	AGCGAGGTATAGAGTTCCTACGT		+									NA
37	miR-289	AGTCGCAGGCTCCACTTAAATATTTA	+	+	+	+	+	+	+	+	+	+	+
38	let-7a	TACTATACAACCTACTACCTCA	+	+	+	+	+	+	+	+	+	+	+
39	miR-100	CACAAGTTCGGATTTACGGGTT	+	+	+	+	+	+	+	+	+	+	+
40	miR-8	GACATCTTTACCTGACAGTATTA	+	+	+	+	+	+	+	+	+	+	+
41	bantam	AATTAGCTTTCACAATGATCTCA	+	+	+	+	+	+	+	+	+	+	+
42	miR-200b	CATCTTTACCTGACAGTATTAGA	+	+	+	+	+	+	+	+	+	+	+
43	miR-2a	GCTCATCAAAGCTGGCTGTGATA	+	+	+	+	+	+	+	+	+	+	+
44	miR-317	ACTGAGATACCACCAGCTGTGTTCA	+	+	+	+	+	+	+	+	+	+	+
45	miR-79	ATGCTTTGGTAATCTAGCTTTA	+	+	+	+	+	+	+	+	+	+	+
46	miR-34b	CAACCAGCTAACCACACTGCCA	+	+	+	+	+	+	+	+	+	+	+

**Total number of expressed miRNAs**			**36**	**35**	**19**	**19**	**22**	**19**	**22**	**19**	**15**	**40**	**36**

All expressed miRNAs confirmed by microarray and Northern blotting methods are summarized in this table. A normalized signal value ≥1,000 was regarded as the positive expression threshold. Nevertheless, only those having signals higher than 1,000 in at least four specimens are listed here as the confirmed members. The same tissues from females and males are unified here. The alpha-numeric order in the left most column is listed sequentially for easier comparison of the results in this file with the descriptions in the text. Abbreviations: +, expressed; -, not expressed; No. the alpha-numeric number; HD, head; BW, body wall; ASG, anterior silk gland; PSG, posterior silk gland; MG, midgut; FB, fat body; OV, ovary; TE, testis; HC, hemocyte; MT, malpighian tubule; Nor, Northern blotting; NA, not assayed.

**Figure 1:**
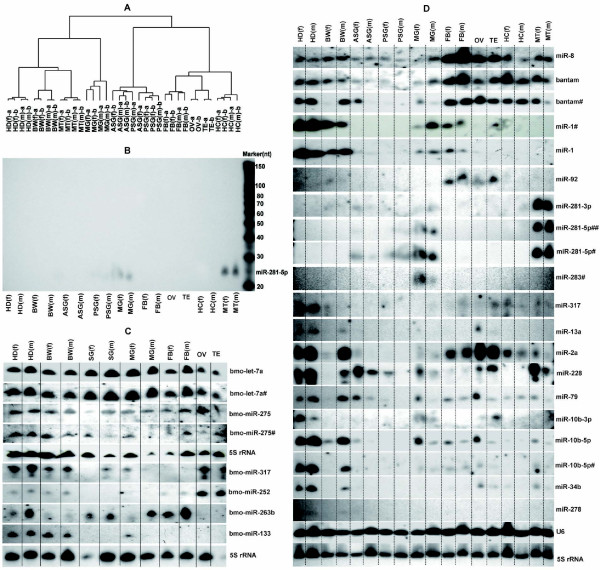
**Spatial expression patterns of miRNAs in fifth-instar day 3 larvae**. (A) Hierarchical clustering of miRNAs based on the normalized microarray signal values in different tissues. The highest signal was 79,375.67 (miR-1 in female head), and the average signal was 3,228.516. Only miRNAs presenting signals higher than 1,000 in at least two specimens were saved as positive and subjected to cluster analysis. In all, 67 unique miRNAs were finally selected as 'confirmed.' CLUSTER 3.0/TreeView software was used for clustering analysis with the median center in array spots. (B), (C) and (D)Northern blotting analysis of miRNAs in multiple tissues of fifth-instar day 3 larvae. (B) Decade Markers (Ambion) were applied in our Northern blotting experiments to estimate miRNA sizes. (C) Initially, we only examined 12 tissues using Northern blotting, and investigated the whole silk gland. (D) For a better comparison with microarray results, we examined the 18 tissues (organs) corresponding to those examined by microarray. The signal bands displaying sex differences are boxed, and those showing some differences from microarray results are underlined. 5 S rRNA and U6 were applied as loading controls. Abbreviations: HD, head; BW, body wall; ASG, anterior silk gland; PSG, posterior silk gland; MG, midgut; FB, fat body; OV, ovary; TE, testis; HC, hemocyte; MT, malpighian tubule; f, female; m, male; TN, tissue number. lowercases 'f' and 'm' in parentheses of (A) are female and male, respectively; 'a' and 'b' represent the average signals of each probe printed at three points on individual blocks.

Hierarchical clustering based on expression variations revealed a clear split between different tissues and organs (Figure 1A). The anterior and posterior silk glands were tightly clustered, but clearly distinct from other tissue subsets, indicating no evident expression differences between the two distal segments of the silk gland. Fat body, gonads and hemocytes from both sexes were closely linked, but clearly separate from each other in clustering. The same tissues and organs from both sexes were obviously clustered into one subgroup, indicating that roles of individual miRNAs could be elucidated by linking to the biology of tissues in which they were uniquely expressed. Northern-blot results confirmed the significant diversity of the spatial expression profiles (Figures 1B, C, and 1D). For instance, both methods confirmed exclusive expression of miR-281-5p and miR-281-3p in the anterior-posterior silk glands, midgut and malpighian tubules of both females and males. This tissue-specific distribution indicates that miRNAs are unlikely to be transported among different tissues, although DNA may travel from the silk glands to the fat body [33].

The silk glands of *B. mori *are completely formed at the end of embryonic development, and highly differentiated into anterior, middle and posterior sections [34,35]. The silk glands are responsible for the production of two categories of cocoon protein, fibroins and sericins. Expression of all sericin genes is limited to the anterior and middle parts of the silk gland [36,37], whereas fibroin genes are expressed exclusively in the posterior silk gland [38,39]. The repeated turn-off and turn-on of sericin and fibroin genes has been attributed to the action of ecdysone and juvenile hormone [34]. In this study, only 19 unique miRNAs were detected in the silk gland, and no section-specific miRNAs were identified using microarrays (Table 1), but Northern-blot analysis revealed significantly higher levels of bantam, miR-228 and miR-79 in the anterior silk gland compared with the posterior silk gland (Figure 1D).

### Sex-biased expression of miRNAs

We screened miRNAs displaying sex-dependent expression patterns using microarray analysis (Table 2). Approximately 20 miRNAs were significantly expressed in the body wall of males (e.g., miR-2a, miR-34b and miR-10b-5p/3p). Additionally, Northern-blot results confirmed sex differences in body wall expression of 10 miRNAs (e.g., bantam, miR-1, miR-13a and miR-2a) (Table 2, Figure 1D). Even though no miRNAs presented obvious section-specific status in the silk gland on the basis of microarray analysis, several miRNAs showed some sex-dependent accumulation in this organ (Table 2, Figure 1D). Importantly, sex-dependent expression was tissue-specific. For example, miR-1 was predominantly expressed in the body wall and fat body in males, and in the malpighian tubules in females. Sex-dependent expression of miR-275 was also observed in the gonads and hemocyte on microarray blocks with two different probes. In all, microarray analyses revealed sex-specific differences in the expression patterns of 16 other miRNAs in the gonads. Specifically, 13 were significantly expressed in the ovaries (e.g., miR-34b, miR-275, miR-305, and let-7a) and four in the testes (miR-252, miR-29b, miR-7, and miR-228,). Northern blotting revealed marked individual differences in the levels of miR-92, miR-317, miR-228, miR-79, miR-10b-3p/5p and miR-34b in the gonads (Figure 1D). However, the sex-bias of miR-92, miR-79 and miR-10b-3p was in contrast with microarray results. Small expression differences in miR-274 were observed in male and female anterior silk glands (1.6-fold) and posterior silk glands (1.6-fold), but differences were more significant in the malpighian tubules (8.7- and 7.3-fold) (Table 2). Based on microarray analysis, 10 female-biased miRNAs were detected in the malpighian tubules (miR-286, miR-228, miR-274, miR-1, miR-252, let-7a, miR-8, bantam, miR-200b, and miR-2a), whereas miR-276-5p and miR-305 showed male-prone accumulation in malpighian tubules. Similar results were obtained by Northern blot analysis for bantam, miR-2a, miR-228 and miR-79 (Figure 1D). miR-263b and miR-133 expression patterns were not significantly different between sexes in microarray analyses. In contrast, Northern blotting confirmed high miR-263b expression levels in the head, midgut and fat body of males, and high miR-133 expression levels in the midgut of females (Figure 1A). Although we cannot exclude a technical basis for this discrepancy, the inconsistent data obtained with microarray and Northern-blot analyses may have arisen from individual differences in the metabolic states of the silkworms used for sampling. This is because some sex-dependent miRNAs have been shown to respond sensitively to nutritional status, which reduces the sex difference, even under mild starvation conditions [40].

**Table 2:** Sex-biased expression of miRNAs

Tissue	Microarray results				Northern results	
	**Female-biased**		**Male-biased**		**Female-biased**	**Male-biased**
				
	**miRNAs**	**Ratio**	**miRNAs**	**Ratio**		

HD						miR-34b
						miR-263b

BW			bantam	3.3, 2.7		bantam
			miR-1	5.3, 6.2		miR-1
			miR-13a	2.1, 1.6		miR-13a
			miR-2a	2.9, 2.2		miR-2a
			miR-228	2.0, 1.4		miR-228
			miR-79	3.5, 3.1		miR-79
			miR-10b-3p	3.1, 3.2		miR-10b-3p
			miR-10b-5p	2.5, 2.7		miR-10b-5p
			miR-34b	2.3, 1.9		miR-34b
			miR-278	2.0, 1.3		miR-278
			miR-252	3.8, 3.4		
			miR-237	2.1, 1.7		
			miR-31a	4.5, 4.2		
			miR-286	2.2, 2.6		
			miR-1497	2.0, 1.7		
			miR-276-3p	2.7, 1.7		
			miR-276-5p	2.8, 2.0		
			miR-275	2.3, 1.8		
			miR-124	2.8, 2.0		
			miR-8	4.4, 3.0		
			miR-184-3p	3.6, 3.4		
			let-7a	2.4, 2.0		
			miR-100	4.1, 4.0		

ASG	bantam	1.5, 1.5	miR-289	1.8, 1.8	bantam	
	miR-79	1.2, 1.3			miR-79	
	miR-281-3p	1.4, 1.4			miR-281-3p	
	miR-281-5p	1.4, 1.4			miR-281-5p	
	miR-228	1.4, 1.4			miR-228	
	miR-274	1.6, 1.7			miR-2a	
	miR-8	1.7, 1.7				

PSG	miR-228	1.4, 1.4	miR-34b	1.8, 1.7	miR-228	
	miR-79	1.2, 1.3			miR-79	
	bantam	1.6, 1.2				

MG					miR-10b-3p	
					miR-10b-5p	

FB			miR-1	3.1, 2.2	miR-79	miR-228

GN	miR-34b	1.6, 1.8	miR-228	3.3, 3.5	miR-10b-5p	miR-92
	miR-10b-5p	1.4, 2.4	miR-7	5.5, 4.5	miR-34b	
	miR-10b-3p	2.3, 2.5	miR-29b	7.9, 6.8		
	miR-275	3.3, 3.4				
	miR-305	2.1, 2.0				
	let-7a	1.7, 2.5				
	miR-307-5p	5.3, 4.9				
	miR-8	1.7, 2.2				
	miR-92	9.1, 10.5				
	miR-9a	2.2, 2.4				
	miR-276-3p	1.7, 1.8				
	miR-317	1.7, 2.4				
	miR-184-3p	1.6, 1.7				

HE	let-7a	1.8, 1.5	miR-275	2.4, 2.3	miR-2a	
			miR-34b	1.6, 1.7	miR-8	

MT	bantam	1.2, 1.5	miR-277	1.3, 1.5	bantam	
	miR-2a	1.4, 1.5			miR-2a	
	miR-228	1.3, 1.4			miR-228	
	miR-274	8.7, 7.3			miR-79	
	miR-286	2.9, 2.1				
	miR-200b	1.7, 2.2				
	miR-8	2.9, 4.6				
	let-7a	1.6, 1.8				
	miR-31a	2.5, 3.1				
	miR-1	1.6, 1.9				

Listed in this table are miRNAs showing sex-bias in any tissue examined. The degree of sex difference is represented by the ratio of the normalized signals between females and males. Abbreviations: HD, head; BW, body wall; ASG, anterior silk gland; PSG, posterior silk gland; MG, midgut; FB, fat body; GN, gonads; HC, hemocyte; MT, malpighian tubule.

Recent studies have also shown sex-related miRNA expression patterns in the rat [40-42]. For example, miR-29b is predominantly expressed in the liver of female rats [40], in contrast to the sex differences observed in silkworm gonads. Specific protein-coding genes of *B. mori *also display sexual differences [43,44]. The livers of rat and human males contain higher levels of genes involved in fuel metabolism, whereas fatty acid translocase is predominantly transcribed in females [45]. Several sex-dependent hepatic genes in the rat liver are regulated by growth hormone (GH) [46,47]. GH significantly down regulates sex-dependent hepatic miRNAs (miR-451 and miR-29b), but does not affect the levels of 5 S rRNA [40]. Cell-cycle genes are also activated during the initiation of midgut metamorphosis, and depend on ecdysone signaling. These results demonstrate multiple new connections between the ecdysone regulatory network and other well-characterized regulatory networks.

### Dynamic expression patterns in tissues during metamorphosis

**Figure 2:**
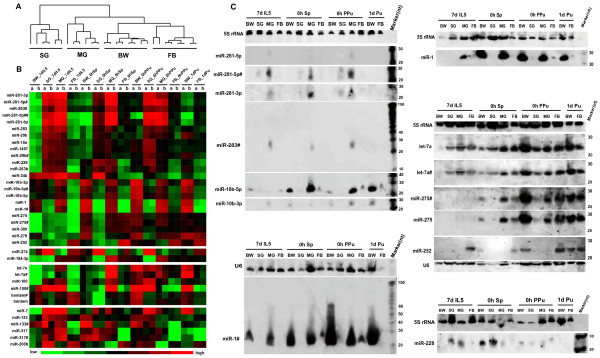
**Spatiotemporal expression of miRNAs in tissues undergoing metamorphosis**. (A) Hierarchical clustering analysis of miRNA expression in four tissues undergoing metamorphosis from larval to pupal stages. Only miRNAs presenting signals higher than 1,000 in at least six specimens were scored as positive and subjected to cluster analysis. In all, data from 83 miRNAs were finally scored as expressed. Clustering analysis was also performed by CLUSTER 3.0/TreeViewsoftware based on array spot median center. (B) miRNAs were obviously differentially expressed in these tissues in microarray analysis. The colors represent relative expression for each miRNA, specifically, green, low; black, mean; and red, high. (C) Northern blotting analysis of miRNAs in four tissues undergoing metamorphosis. 5 S rRNA and U6 served as loading controls. Both microarray and Northern blotting analyses with alternative probes for bmo-miR281-5p, bmo-miR-10b-5p, bmo-miR-1, bmo-let-7a and bmo-miR-275 yielded reproducible results. Abbreviations: 7d IL5, Fifth-instar day 7 larvae; 0hr Sp, 0 hour spinning larvae; 0hr PPu, 0 hour prepupae; 1d Pu, day 1 pupae; HD, head; BW, body wall; SG, silk gland; MG, midgut; FB, fat body; 'a' and 'b' represent the average signals of individual probes printed at three points on each block.

To determine the extent of tissue-specific changes during specific developmental events, we assessed changes in miRNA expression in four individual tissues and organs (body wall, silk glands, midgut and fat body) from fifth-instar day 7 larvae to pupae. The microarray raw data and processed files are also accessible through GEO Series accession number GSE18039. To make the inter-slide signals more comparable, all net signals of scanned points were normalized using a global-mean method. The unsupervised hierarchical clustering of miRNA expression based on microarray clearly separated these four tissues (Figure 2A), with greater differences in miRNA expression between tissues than developmental stages. Both methods confirmed that most miRNAs were preferentially expressed in individual tissues (Figures 2B and 2C). For example, miR-281 and miR-283 were mainly observed in the silk glands and midgut. Alternative probes used with both methods confirmed substantial miR-1 expression levels in the body wall and midgut during metamorphosis, which was clearly observed in the fat body of cocoon-spinning larvae and lasted until the end of the spinning period. miR-34b was highly expressed in the four tissues of late fifth instar larvae, and exclusively induced in the silk glands at the onset of the wandering stage; this was followed by a sharp decrease in expression in the four tissues at the end of the cocoon-spinning period. In contrast to miR-34b, low levels of miR-275 and miR-305 were confirmed with both methods in the four tissues of fifth-instar day 7 larvae, but strong expression was verified in the body wall, midgut and fat body from the spinning larval to pupal stages. Notably, miRNA-274 expression was exclusively detected in the silk glands during metamorphosis. Complementary to miR-274 expression, miR-184-3p was significantly expressed in the body wall, midgut and fat body, but was absent from the silk glands. miR-252 was most strongly detected in the fat body compared with other tissues during metamorphosis. The two methods also showed that miR-228 was almost undetectable in the body wall of fifth-instar day 7 larvae and in the fat body during the whole metamorphosis period, but was highly expressed in the histolytic silk glands and midgut. Some other miRNAs displaying tissue-prominent accumulation are included in Figure 2.

We were particularly interested in miRNAs that are developmentally regulated within individual tissues. Accordingly, we compared the expression patterns of these miRNAs in each tissue during metamorphosis. The expression levels of 63 miRNAs were significantly altered in at least one of the four tissues during the developmental transition from larvae to pupae (Additional file 1). A small fraction of miRNAs increased or decreased expression in the four tissues, indicative of common responses in shared functions. Common or similar regulatory motifs and cognate transcription factors may be responsible for such co-expression. Among these miRNAs, miR-275 and miR-305 are clustered in the silkworm genome [30] and were found to be coordinately upregulated. In addition, ten miRNAs increased and eight miRNAs decreased in at least two tissues. An extremely small set of miRNAs displayed unique alterations in each particular tissue. Only 14 miRNAs showed divergent expression patterns, with increasing abundance in one tissue and decreasing abundance in another. The larval-specific tissues, silk glands and midgut, contained a larger set of downregulated miRNAs during apoptosis, whereas the body wall and fat body had a larger number of upregulated miRNAs during metamorphic transitions. Although miR-2a and miR-13a, miR-10a and miR-10b are closely clustered in the silkworm genome [30], their expression patterns were distinct. Such discrepancies between the expression profiles of unique miRNAs may be attributed to the existence of separate *cis*-regulatory controls. In general, these results provide a record of the set of miRNAs that are differentially expressed during the onset of metamorphosis.

Experiments with alternative probes on females and males confirmed upregulation of let-7 in the body wall, silk glands and fat body, but some fluctuations in the midgut; its expression changes in these tissues further support its potential roles in histolysis and histogenesis [29]. bantam miRNA exhibited constant expression levels in the body wall, silk glands and midgut, but presented significant down-regulation in the fat body (Additional file 1). miR-274 expression was restricted to the silk glands and malpighian tubules (Table 1), and dramatically declined in the silk glands during metamorphosis (Additional file 1). Previous studies have shown robust miR-274 expression during the vigorous periods of silk gland growth and spinning [30], suggesting it has a role in silk gland regulation.

During *B. mori *development, almost all tissues and organs undergo different stages of metamorphic transformation. In the final larval stage, tissues are pupally committed, and their developmental fate is restricted to pupal differentiation under the action of ecdysteroid secreted during the prepupal period [48-50]. The silkworm midgut and silk glands are committed to degenerate after pupal ecdysis within about 48 hours [51-53]. The fat body, however, survives into adulthood through simultaneous histolysis and histogenesis in each transition, particularly from larva to pupa [54]. Death or renewal of these tissues is triggered by ecdysone [48,55,56] and coordinated by the miRNA pathway [12,57]. The physiological functions of miRNAs in histolysis and histogenesis during silkworm metamorphosis are currently open to speculation. Investigation of miRNA expression in these tissues under conditions of programmed cell death or apoptosis may aid in establishing the critical molecular mechanisms underlying metamorphosis.

### Differential or coordinate expression of miRNA arms as well as sense and antisense transicripts

In the present study, we observed that miRNAs from two arms of the precursor were not always equally transcribed (see Additional files 2A, B). For example, in the fifth-instar day 3 larvae, miR-307-5p was expressed at greatest levels in hemocytes, followed by the head, body wall, fat body, ovaries and malpighian tubules for both sexes, but miR-307-3p was not detected in all examined tissues. miR-iab-4-5p was widely expressed in the body wall, midgut, fat body, ovaries, testes and malpighian tubules in both sexes, but only appeared in the body wall of males. Interestingly, miR-276-5p was only expressed in the malpighian tubules of females, whereas miR-276-3p was ubiquitously expressed in all tissues other than the testes. However, miR-281-5p and miR-281-3p were strongly expressed in the malpighian tubules, silk glands and midgut, and weakly expressed in the head and body wall. Conversely, miR-10b-5p and miR-10b-3p displayed strong expression in the head and relatively weak expression in the body wall, midgut and malpighian tubules in both sexes, but were almost undetectable in the silk glands, fat body, gonads and hemocytes. In tissues undergoing histolysis and regeneration, miR-10b-3p/5p pair strongly expressed in the body wall and midgut, and miR-281-3p/5p pair most strongly expressed in the silk gland and midgut; here miRNAs from both arms demonstrated a high degree of co-expression. Nevertheless, other 5p/3p pairs showed large differences during this stage of metamorphosis (Additional file 2B). The underlying mechanism responsible for the co-expression is elusive since this coordinate accumulation occurred selectively.

We also used the sense probes of four miRNAs to examine their antisense transcripts. The asymmetric expression observed for miRNAs was also evident for sense and antisense miRNAs (Additional files 2C, D). In detail, miR-276-5p was only expressed in the malpighian tubules of female fifth-instar day 3 larvae, but its antisense strand was detected in the body wall and malpighian tubules of both sexes. Similarly, miR-263b was only expressed in the head of the two sexes but its antisense strand was detected in the body wall of males. miR-2a was ubiquitously expressed in all tissues, with the strongest signals found in the head and body wall, followed by the gonads and malpighian tubules, and then the silk glands, fat body and midgut, but the antisense strand was not found in these tissues. During metamorphosis, bmo-miR-2a was highly expressed in the four tissues, but no expression signals were detected for its antisense transcript; both miR-276-5p and its antisense transcript were absent or expressed at low levels in the body wall, and fat body, but strongly accumulated in the silk gland and midgut. Their expression changed in a coordinate way, but, according to microarray analysis, miR-276-5p tripled in the silk gland throughout the entire spinning stage, in comparison with anti-miR-276-5p. miR-124 and its antisense transcript showed a high degree of co-expression in the four tissues of fifth-instar day 7 larvae, but expression differences appeared in the larvae at the onset of cocoon spinning, and became greater in the following stages. Sense/antisense miRNAs seem to be employed in a wide range of species [58]. We speculate that more loci in the silkworm will be found to produce sense/antisense miRNAs from the noncoding DNA strand since the reverse complements of many other pre-miRNA hairpins also fold into hairpins reminiscent of miRNA precursors.

## Conclusions

Here, we describe the first spatial expression profile of miRNAs in the silkworm. The diverse miRNA expression patterns we observed indicate that specific aspects of spatial patterning are subject to regulation by miRNAs. Furthermore, we observed varying spatiotemporal patterns of miRNA expression in the silkworm from the larval to pupal stages. Integration of fifth instar day 3 larval and spatiotemporal expression data during tissue metamorphosis should aid in guiding future functional studies of these miRNAs.

## Methods

### Silkworm culture and RNA extraction

A bivoltine strain (DaZao) of *B.mori *was used in this study. After hatching, larvae were reared on mulberry leaves at 25-27°C and 85% H.R. under a 12 h light/12 h dark photoperiod and harvested at specific developmental stages. To generate comprehensive spatial profiles of miRNAs, we collected 18 tissue specimens from both sexes of fifth-instar day 3 larvae, specifically, HD, BW, ASG, PSG, MG, FB, OV and TE, HC, MT. Additionally we collected BW, SG, MG and FB from the late larval to pupal stages. Total RNA was individually isolated from different tissues of silkworms using TRIzol (Invitrogen, USA).

### Microarray printing and hybridization

To determine the global spatial distribution of miRNAs, we designed a DNA oligonucleotide-based microarray, which was constructed largely as described previously [30]. The miRNA probes (designated 'SW' followed by a serial number) on the microarray were complementary to the mature sequences of miRNAs, concatenated up to 40 nt with polyT, and modified with an amino group at the 5'-end. Since probe sets for some miRNAs were present more than once on the array, 106 probes in all were used to investigate the spatial profiling of the 92 unique silkworm miRNAs [30]. These comprised 45 probes for the miRNAs identified by homology searching, 2 for silkworm-specific miRNAs, 4 for the antisense strands of miRNAs, 42 for miRNAs of other organisms non-homologous to the silkworm genome, and 13 as replicate probes for several miRNAs. All probes were synthesized at MWG Biotech (Ebersberg, Germany), dissolved in EasyArray spotting solution (CapitalBio, Beijing, China) at a concentration of 40 μmol/L, and printed in triplicate on aldehyde-coated slides (CapitalBio) using a SmartArray-136 spotter (CapitalBio). Low molecular weight RNA (4 μg) isolated using PEG solution precipitation was labeled using fluorescent Cy3 with T4 RNA ligase, according to a previous protocol [59], and hybridized overnight to the microarray in 16 μl hybridization buffer (15% Formamide, 0.2%SDS, 3 × SSC, 50 × Denhardt's) at 42°C. After hybridization, a SlideWasher-8 instrument (CapitalBio) was applied to wash the slides using washing buffer I(0.2%SDS, 2 × SSC) and buffer II (0.2 × SSC). Slides were dried and then scanned using a laser confocal scanner, LuxScan 10K-A, and images were extracted from data using LuxScan 3.0 software (CapitalBio). Net signals were calculated by subtracting the local background from total intensities, and spots with a negative signal were assigned the value 10. To make the inter-slide signals comparable, net signals of fifth-instar day 5 larvae were normalized on the basis of U6 and 5 S rRNA signals, and net signals of tissues during metamorphosis were normalized with a global-median method [60]. Flawed spots were excluded for further analysis after visual inspection of the hybridization figures using a self-developed program, "Flaw-Spot-Finder," according to X and Y axes of the spot position on the array. The differentially expressed miRNAs were selected with Significance Analysis of Microarrays (SAM, version 3.0) as described previously [61,62]. Signal values of every triplicate spot for each probe on a slide were averaged, and each sample was hybridized with two replicate slides designated 'a' and 'b.' The mean signal values were log_2 _transformed before submission to Gene Cluster 3.0 for cluster analysis. Microarray data passing a threshold of 1000 is generally confirmed by Northern blotting. Therefore, the signal value '1000' was set as the positive expression threshold. Net normalized signal values from each sample were classified into four grades for better analysis. Specifically, values lower than 1000 represented 'not expressed'; 1000 to 4999 represented 'normally expressed'; 5000 to 9999 represented 'highly expressed'; and higher than 10000 were 'excessively expressed', since the saturated intensity on the array was 65,535. The raw microarray data and processed files have been deposited in the NCBI Gene Expression Omnibus [31] under accession number GSE18039. http://www.ncbi.nlm.nih.gov/geo/query/acc.cgi?acc=GSE18039.

### Northern blotting

Total RNA (150 μg) was fractionated using a denaturing 12% polyacryamide-7 mol/L urea gel. RNA contained within the gel was electroblotted to Hybond-N nylon membranes (Ambion) using semi-dry Trans-Blot Electrophoretic Transfer (Bio-Rad). After transferring, RNA was fixed to the membrane by UV cross-linking (1000 μJ, HL-2000 HybriLinker; UVP), followed by baking in a vacuum oven at 80°C for 30 min. DNA oligonucleotides complementary to miRNAs, U6 RNA and 5 S rRNA, were synthesized (Sangon, Shanghai). The 5'-ends of the DNA and Decade Markers (Ambion) were labeled with [γ-^32 ^P] ATP (Amersham) using T4 polynucleotide kinase (Takara) and subjected to purification using a Purification Cartridge (Ambion). The membrane was pre-hybridized in prehybridization solution containing 6 × SSC, 10 × Denhardt's solution, 0.2% SDS and 30 μg salmon sperm DNA (Ambion) at 65°C for about 5 h. Membranes were hybridized in hybridization solution containing 6 × SSC, 5 × Denhardt's solution, 0.2% SDS and 50 μg denatured sheared salmon sperm DNA (Ambion) with 1-5 × 10^6 ^cpm eluted radiolabeled oligonucleotide probes at 10-15°C below the calculated dissociation temperature for at least 10 h. Blots were washed three times for 5 min each at 37°C with 6 × SSC and 0.2% SDS and once at 42°C for at 15 min. After the final wash, blots were wrapped in plastic film and exposed to X-ray film at -70°C for the appropriate time. Finally, the former probes on the Hybond-N nylon membranes were stripped off by washing three times at 90°C in 0.1 × SSC, 0.5% SDS.

## Abbreviations

*B.mori*: *Bombyx mori*; miRNA: microRNA; 3'UTRs: 3'untranslated regions; HD: head; BW: body wall; SG: silk gland; ASG: anterior silk gland; PSG: posterior silk gland; MG: midgut; FB: fat body; OV: ovary; TE: testis; HC:hemocyte; MT: malpighian tubule; GN: gonad; f: female; m: male; SAM: Significance Analysis of Microarrays.

## Authors' contributions

SPL conceived and designed the study, performed microarray and Northern blotting hybridization, analyzed data, and wrote the manuscript. SG, DYZ and JYY prepared the samples and extracted RNAs. QYX and ZHX coordinated the study. QYX reviewed the manuscript. All authors have read and approved the final manuscript.

## Supplementary Material

Additional file 1 **Tissue- and organ-specific changes in miRNA expression**. Normalized data of expressed miRNAs were submitted to SAM 3.0 for time-course analysis. miRNAs displaying downregulation or upregulation in all tissues during metamorphosis are presented in light blue. miRNAs displaying downregulation or upregulation in at least two tissues are depicted in light purple, while those downregulated or upregulated in only one tissue are colored gray. The miRNAs colored light yellow are upregulated in one tissue, but downregulated in another tissue. Only miRNAs up- or downregulated in at least one tissue are presented. Abbreviations: BW, body wall; SG, silk gland; MG, midgut; FB, fat body; reg, regulated; score (d), the T-statistic value; up, upregulated; down, downregulated. Click here for file

Additional file 2 **Microarray-based analyses of coordinate transcription and asymmetric transcription of miRNAs**. Co-expression was usually observed for miRNAs derived from both arms of the same precursors, as well as for some miRNAs generated at either strand of the same locus. (A) and (B) Not all miRNAs from both arms of the precursors were coordinately expressed; (C) and (D) likewise, not all miRNAs from both strands of the same locus were coordinately accumulated. (A) Comparison of 3p/5p miRNA pairs in multiple tissues of fifth-instar day 3 larvae. (B) Comparison of 3p/5p miRNA pairs in the four tissues undergoing metamorphosis from larval to pupal stages. (C) Comparison of sense and antisense miRNAs in multiple tissues of fifth-instar day 3 larvae. (D) Comparison of sense and antisense miRNAs in the four tissues undergoing metamorphosis. Abbreviations: HD, head; BW, body wall; ASG, anterior silk gland; PSG, posterior silk gland; MG, midgut; FB, fat body; OV, ovary; TE, testis; HC, hemocyte; MT, malpighian tubule; f, female; m, male; 7d IL5, fifth-instar day 7 larvae; 0hr Sp, 0-hour spinning larvae; 0hr PPu, 0-hour prepupae; 1d Pu, day 1 pupae; 'a' and 'b' represent the average signals of each probe printed at three points on individual blocks. Click here for file
