# Supplementary material for: MicroRNAs show diverse and dynamic expression patterns in multiple tissues of Bombyx mori
Source: BMC Genomics. 2010 Feb 2;11:85. doi: 10.1186/1471-2164-11-85 (PMC2835664; doi:10.1186/1471-2164-11-85)
Supplement: Additional file 1 — Tissue- and organ-specific changes in miRNA expression. Normalized data of expressed miRNAs were submitted to SAM 3.0 for time-course analysis. miRNAs displaying downregulation or upregulation in all tissues during metamorphosis are presented in light blue. miRNAs displaying downregulation or upregulation in at least two tissues are depicted in light purple, while those downregulated or upregulated in only one tissue are colored gray. The miRNAs colored light yellow are upregulated in one tissue, but downregulated in another tissue. Only miRNAs up- or downregulated in at least one tissue are presented. Abbreviations: BW, body wall; SG, silk gland; MG, midgut; FB, fat body; reg, regulated; score (d), the T-statistic value; up, upregulated; down, downregulated. [file 1471-2164-11-85-S1.DOC]

| **miRNA** | **BW** | | **SG** | | **MG** | | **FB** | |
| --- | --- | --- | --- | --- | --- | --- | --- | --- |
|  | **regulated** | **score(d)** | **regulated** | **score(d)** | **regulated** | **score(d)** | **regulated** | **score(d)** |
| miR-275# | up | 1.34 | up | 0.98 | up | 1.88 | up | 1.01 |
| miR-275 | up | 1.23 | up | 0.68 | up | 1.71 | up | 0.95 |
| miR-305 | up | 0.99 | up | 1.14 | up | 2.33 | up | 1.22 |
| miR-31a | up | 1.81 | up | 1.53 | up | 7.88 | up | 2.33 |
| miR-279 | up | 1.34 | up | 1.32 | up | 10.72 | up | 3.91 |
| miR-2a | up | 0.79 | up | 1.45 | up | 2.88 | up | 1.09 |
| let-7a | up | 2.05 | up | 1.37 |  |  | up | 1.31 |
| let-7a# | up | 2.7 | up | 1.57 |  |  | up | 1.00 |
| miR-276-5p | up | 1.18 | up | 4.08 |  |  |  |  |
| miR-276-3p |  |  | up | 2.5 |  |  | up | 2.93 |
| miR-252 | up | 1.62 |  |  | up | 3.55 |  |  |
| miR-315 | up | 1.21 |  |  | up | 1.00 |  |  |
| miR-1 | up | 0.56 |  |  | up | 0.26 | up | 1.03 |
| miR-1# | up | 1.03 |  |  | up | 3.26 | up | 0.55 |
| miR-10b-3p | up | 1.3 |  |  |  |  | up | 1.18 |
| miR-10b-5p# | up | 1.28 |  |  |  |  | up | 1.60 |
| miR-307-5p | up | 1.55 |  |  |  |  | up | 1.42 |
| miR-184-5p | up | 1.25 |  |  |  |  | up | 1.11 |
| miR-228 | up | 1.18 |  |  |  |  |  |  |
| miR-29b | up | 2.01 |  |  |  |  |  |  |
| miR-29b# | up | 3.73 |  |  |  |  |  |  |
| miR-92 | up | 1.54 |  |  |  |  |  |  |
| miR-283 | up | 2.22 |  |  | down | -0.92 |  |  |
| miR-263b | up | 1.74 |  |  | down | -1.54 |  |  |
| miR-87 | up | 1.26 |  |  | down | -2.18 | up | 1.12 |
| miR-929 | up | 1.03 |  |  | down | -1.04 |  |  |
| miR-100# | up | 1.11 |  |  | down | -4.26 |  |  |
| miR-100 | up | 1.29 |  |  | down | -0.72 |  |  |
| miR-237 | up | 1.1 |  |  | down | -1.18 |  |  |
| miR-10a | up | 1.65 |  |  | down | -1.86 |  |  |
| miR-1497 | up | 1.23 |  |  | down | -1.59 |  |  |
| miR-iab-4-3p | up | 1.22 |  |  | down | -1.01 |  |  |
| miR-289 | up | 1.22 | up | 1.67 | down | -1.03 | up | 1.24 |
| miR-124 | up | 3.08 | down | -2.13 |  |  | up | 1.13 |
| miR-184-3p | up | 1.66 | down | -1.19 |  |  | down | -1.74 |
| miR-7 | up | 2.43 | down | -0.57 | down | -1.79 |  |  |
| miR-308 |  |  | down | -1.33 |  |  | up | 1.27 |
| anti-miR-124 |  |  | down | -1.69 | down | -4.15 |  |  |
| miR-8 |  |  | down | -1.12 | down | -2.41 |  |  |
| miR-310 |  |  | down | -1.7 | down | -2.17 |  |  |
| miR-313 |  |  | down | -1.02 | down | -4.96 |  |  |
| miR-274 |  |  | down | -0.94 |  |  |  |  |
| miR-286 |  |  | down | -1.22 |  |  |  |  |
| miR-9b |  |  | down | -2.25 |  |  |  |  |
| miR-309 |  |  | down | -2.48 |  |  |  |  |
| miR-287 |  |  | down | -2.86 |  |  |  |  |
| miR-14 |  |  | down | -1.00 |  |  |  |  |
| miR-31b |  |  | down | -2.22 |  |  |  |  |
| miR-71 |  |  |  |  |  |  | up | 1.56 |
| miR-12 |  |  |  |  |  |  | up | 2.21 |
| miR-277 |  |  |  |  |  |  | up | 0.83 |
| miR-9a |  |  |  |  |  |  | up | 1.67 |
| bantam |  |  |  |  |  |  | down | -1.35 |
| bantam# |  |  |  |  |  |  | down | -1.13 |
| anti-miR-276-5p |  |  |  |  | down | -3.27 |  |  |
| miR-133# |  |  |  |  | down | -1.52 |  |  |
| miR-5 |  |  |  |  | down | -2.19 |  |  |
| miR-200b |  |  |  |  | down | -6.83 |  |  |
| miR-6 |  |  |  |  | down | -1.19 |  |  |
| miR-278 |  |  |  |  | down | -3.10 |  |  |
| miR-133 |  |  |  |  | down | -2.94 |  |  |
| miR-4 |  |  |  |  | down | -1.45 |  |  |
| miR-306 |  |  |  |  | down | -3.88 |  |  |
| miR-283# |  |  |  |  | down | -2.75 |  |  |
| miR-288 |  |  |  |  | down | -2.05 | down | -1.05 |
| miR-79 |  |  |  |  | down | -1.94 | down | -1.22 |
| miR-317 |  |  |  |  | down | -1.94 | down | -1.17 |
| miR-317# |  |  |  |  | down | -2.00 | down | -1.95 |
| miR-iab-4-5p |  |  |  |  | down | -0.91 | down | -1.13 |
| miR-34b | down | -1.09 | down | -0.80 | down | -3.46 | down | -1.70 |
| **SUM(up/down)** | **up:31/down:1** | | **up:9/down:16** | | **up:8/down:30** | | **up:20/down:7** | |
