# Supplementary figures and images for: MicroRNAs show diverse and dynamic expression patterns in multiple tissues of Bombyx mori
Source: BMC Genomics. 2010 Feb 2;11:85. doi: 10.1186/1471-2164-11-85 (PMC2835664; doi:10.1186/1471-2164-11-85)

A

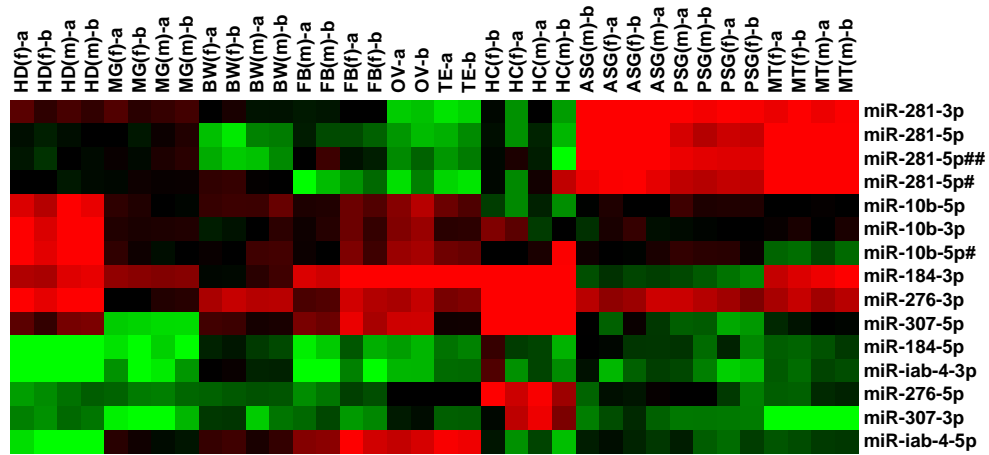

B

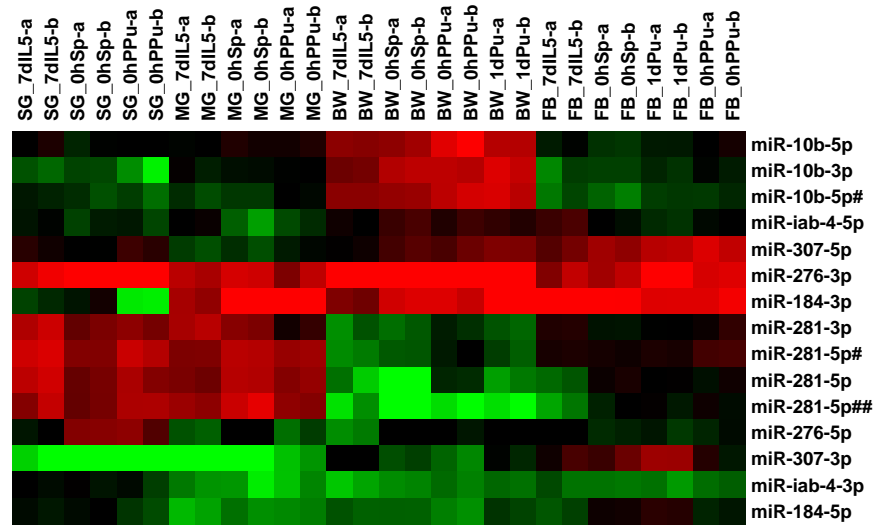

C

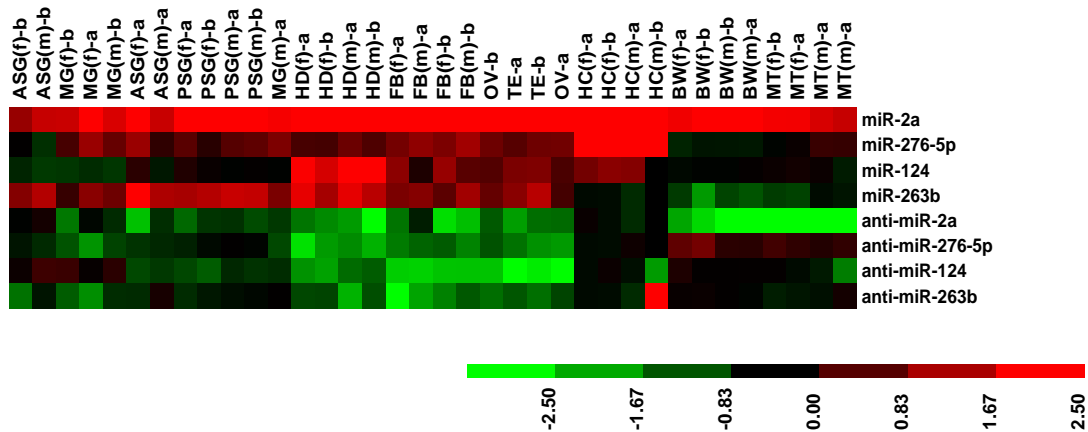

D

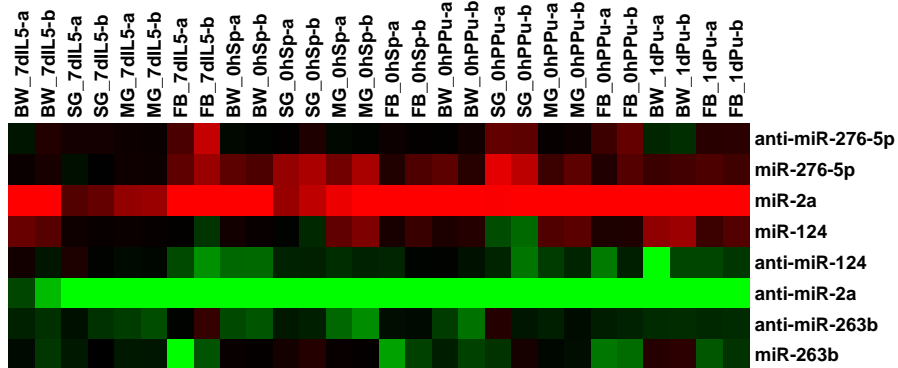

Supplement: Additional file 2 — Microarray-based analyses of coordinate transcription and asymmetric transcription of miRNAs. Co-expression was usually observed for miRNAs derived from both arms of the same precursors, as well as for some miRNAs generated at either strand of the same locus. (A) and (B) Not all miRNAs from both arms of the precursors were coordinately expressed; (C) and (D) likewise, not all miRNAs from both strands of the same locus were coordinately accumulated. (A) Comparison of 3p/5p miRNA pairs in multiple tissues of fifth-instar day 3 larvae. (B) Comparison of 3p/5p miRNA pairs in the four tissues undergoing metamorphosis from larval to pupal stages. (C) Comparison of sense and antisense miRNAs in multiple tissues of fifth-instar day 3 larvae. (D) Comparison of sense and antisense miRNAs in the four tissues undergoing metamorphosis. Abbreviations: HD, head; BW, body wall; ASG, anterior silk gland; PSG, posterior silk gland; MG, midgut; FB, fat body; OV, ovary; TE, testis; HC, hemocyte; MT, malpighian tubule; f, female; m, male; 7d IL5, fifth-instar day 7 larvae; 0hr Sp, 0-hour spinning larvae; 0hr PPu, 0-hour prepupae; 1d Pu, day 1 pupae; 'a' and 'b' represent the average signals of each probe printed at three points on individual blocks. [file 1471-2164-11-85-S2.PDF]
